# Exercise time of day and blood pressure: Considering chronotype for precision health

**DOI:** 10.1038/s41371-024-00929-y

**Published:** 2024-06-27

**Authors:** Steven K. Malin

**Affiliations:** 1 Department of Kinesiology & Health, Rutgers University, New Brunswick, NJ USA; 2 Division of Endocrinology, Metabolism & Nutrition, Department of Medicine, New Brunswick, NJ USA; 3 New Jersey Institute for Food, Nutrition & Health, Rutgers University, New Brunswick, NJ USA; 4 Institute for Translational Science & Medicine, Rutgers University, New Brunswick, NJ USA

**Keywords:** Hypertension, Risk factors

The health benefits of exercise among people at risk for cardiovascular disease (CVD) are well accepted. However, many health professionals and researchers debate the optimal exercise “dose” (e.g. intensity, modality, and/or duration) for improving cardiometabolic health [1, 2]. In recent years, the best time of day to exercise has become a popular and open question, as research has primarily focused on morning versus afternoon timeframes. A practical consideration here is that time is often reported as a major barrier to exercise. Advising people to simply move when they can, seems to be a reasonable recommendation. A trick though for at least many adults relates to weekly work schedules that may limit the ability to dedicate time to exercise in the morning or afternoon. Pre-planning nonetheless can be an advantage when considering the time of day to exercise. Thus, empowering people to select when they feel is best to exercise would be anticipated to lead to long-term adherence. Chronotype is a term in circadian biology used to describe “early birds” and “night owls” with respect to performing various acts of daily living, including eating, physical activity, sleep, and work preferences [3]. But, whether exercise impacts health in relation to chronotype is not clear.

Novel work [4] presented by Boettcher and colleagues in the *Journal of Human Hypertension* demonstrates that exercise during morning, afternoon, or evening times produced similar rises in systolic blood pressure of approximately 62–67 mmHg (i.e. submaximal stage 4 Bruce protocol systolic – pre-exercise systolic) in young healthy adults. This is of interest since resting blood pressure has a circadian rhythm, such that it rises during the morning hours, has slight variations throughout the day, and dips during the sleep period. Whether exercise interacts with underlying circadian biology to influence exercise blood pressure responses is somewhat unclear. These findings [4] suggest that the rise in exercise systolic blood pressure does not differ across different times of day in young adults. Clinically, this is valuable information since exaggerated exercise blood pressure responses (e.g. submaximal exercise, > 170 mmHg) are an independent risk factor for hypertension and cardiovascular disease [4]. Interestingly, the novel study [4] noted that 26% of the young adults (*n* = 8 of 31) had exaggerated exercise blood pressure responses on at least 2 tests. This highlights that while exercise has accepted health benefits, some individuals without known hypertension may have episodes of large blood pressure surges during acts of daily living that place them at higher risks for CVD. Notwithstanding this, the observation that blood pressure rises similarly across the day seems fair given that exercise was performed across graded intensities (i.e. Bruce protocol), and blood pressure during exercise needs to rise in order to promote the delivery of blood to working tissues. Whether the response would be identical in aging adults with and without comorbidities, however, remains to be seen. Regardless, the fact that rises in systolic blood pressure during exercise were similar though across time of day contradicts evidence that morning periods tend to be related to reduced resting endothelial function compared with mid-day and afternoon periods [5]. In turn, a fair question is do all chronotypes respond the same way to exercise? This is important since chronotypes are noted as having unique CVD risk profiles, and this could be masked when averaging results in studies examining such questions around exercise time of day. In fact, we recently showed that earlier chronotype people with obesity who are middle-aged to older adults perform more physical activity throughout the morning hours and have greater daily energy expenditure than later chronotypes [6]. This coincided with general observations that earlier chronotypes tend to be more metabolically insulin sensitive [7], have better vascular function [5], and lower blood pressure in response to exercise [8] when compared with late chronotypes, thereby conferring lower CVD risk. In the current novel work [4], chronotype was assessed by the Morning-Eveningness Questionnaire (MEQ) and people were separated as morning (MEQ score ≥59), intermediate (MEQ score 42–58), or evening (MEQ score ≤41). Interestingly, higher MEQ scores (i.e. favoring morning profiles) were related to exaggerated exercise blood pressure responses during the afternoon and evening tests only. This highlights that chronotype may interact with exercise time of day adaptation.

The finding that exercise timing may matter by chronotype is unique. Although the emphasis on chronotype was not the focus of the current paper [4], the work does showcase that morning chronotypes may have exaggerated exercise systolic blood pressure responses when performed in the afternoon and/or evening. This work aligns with a study in healthy young adults indicating that early chronotype individuals who exercise later in the day can create mismatches of their internal circadian clock to the environmental clock [9]. The long-term ramifications of such circadian misalignment on blood pressure or general health remain to be studied in randomized trials, particularly since the primary outcome here was exercise systolic blood pressure. Indeed, there was no difference in resting blood pressure across time of day [4], nor was the measurement of blood pressure during sleep made. This is important as there is mixed evidence that morning versus afternoon exercise may yield better results for resting blood pressure [2, 10]. In either case, morning exercise has been suggested for the general public due to several reasons, including evidence for better overall exercise adherence and lower caloric intake [11] as well as practically offsetting later in the day responsibilities surrounding work and/or family. This said the present work on exercise blood pressure was during awake hours [4] without considerations of sleep deprivation – a factor commonly observed in people juggling various life responsibilities. Recent work has shown that acute exercise prior to sleep deprivation benefits 24-hr blood pressure primarily in people of late chronotype [12]. This highlights the potential interactions of sleep on modifying exercise-mediated blood pressure responses by chronotype. Nevertheless, the health benefit may be outcome-specific as other work suggests afternoon exercise may be best for glucose regulation [13, 14]. Further, work from the Look AHEAD trial suggested that while males had no differential effect for exercise time of day on aerobic fitness or glycemic control as indicators of cardiovascular health, females were reported to derive more benefit from afternoon exercise [15]. Thus, if exercise timing does matter most by aligning chronotype, and sex could play additional roles, then questions arise with regard to personalizing exercise prescription and synchronizing exercise with individual circadian biology.

Current guidelines do not provide specific guidance on when really to perform exercise for cardiometabolic health. Neither are they specific on what aerobic, or even resistance, exercise means in the context of timing. This later point may be of consideration for the field given often individuals are advised to do both aerobic and resistance exercises for general health. Knowing too that resistance exercise may create surges in blood pressure that can surpass that of aerobic exercise, additional work is merited. Another consideration is the aforementioned work was in young adults. It is known that an individual’s preference for performing acts of daily living adjusts across a given lifespan (i.e. children tend to be earlier chronotypes, adolescence shifts preference toward the evening, and progression to older age reverts back to earlier tendencies). Coupled with existing blood pressure exercise work in young to older adults [4, 8], the literature highlights that time of day may matter for specific chronotypes. Moreover, the collective literature raises consideration for when measures are actually made (e.g. after an overnight fast vs. late in the day) and how that aligns with the underlying biology. Identifying strategies therefore that foster the adoption of healthy lifestyle behaviors early in the diagnosis of CVD is more important than ever since there is no quick fix. Engaging people in exercise has proven difficult in years as evidenced by, in part, rises in obesity and diabetes. Thus, examining exercise timing and chronotype could provide healthcare professionals with better guidance on how to prescribe exercise for long-term well-being.
